# A rare presentation of hydatid cyst in abdominal wall: case report

**DOI:** 10.11604/pamj.2021.40.246.32301

**Published:** 2021-12-21

**Authors:** Leena Moshref, Haifaa Malaekah

**Affiliations:** 1 Department of General Surgery, Dr Soliman Fakeeh Hospital, Jeddah, Kingdom of Saudi Arabia,; 2 Department of General Surgery, Fakeeh College of Medical sciences, Jeddah, Kingdom of Saudi Arabia

**Keywords:** Hydatid cyst, abdominal wall, outcome, case report

## Abstract

Echinococcus species cause hydatidosis, which is a verminosis. Echinococcus vogeli results in polycystic hydatid disease, and wild dogs are the definitive hosts. In fact, wild dogs and rats are the most common hosts. The transit of Echinococcus eggs through the liver and lungs is hypothesized to result in hydatid cyst formation in the subcutaneous tissue. In 1.5 percent of patients with hydatidosis, hydatid cysts of the subcutaneous tissue have been documented. They ranged from 0.6 percent to 2.6 percent. We here report a case of hydatid cyst of the abdominal wall which was surgically treated. A 30-year-old lady had been experiencing pain associated with lump in her right lower abdomen for three months. On clinical examination, an enlargement in the left side measuring 4 x 3 cm was discovered. Imaging was performed preoperatively to rule out other differential diagnoses. Ultrasound was performed, followed by computed tomography and magnetic resonance imaging, which revealed multilocular cystic mass measuring 9 x 8.5 x 4.7 cm along the right lower anterior abdominal wall muscles (with cysts inside cysts), which suggested hydatid cyst. Histopathology confirmed the diagnosis of hydatid cyst, after the mass was surgically removed. Treatment with albendazole was continued. Hydatid cyst of the subcutaneous tissue is uncommon, with only a few occurrences recorded in the literature. This study describes a case of hydatid cyst in an uncommon place. Imaging confirmed the diagnosis, and the tumour was surgically removed. It ruptured during surgery and was successfully treated with hypertonic saline and albendazole. Then it was adequately managed. Given that subcutaneous hydatid cyst is rare, it's vital to keep in mind that it can be a possible cause of abdominal wall mass. Specific imaging test is essential to correctly locate and remove it. It must be treated with anthelmintic before surgery, in order to reduce the risk of contamination due to cyst rupture during surgery. Subcutaneous hydatid cyst should be considered one of the differential diagnoses for soft tissue masses, in particular in patients living in endemic areas. To rule out alternative possibilities, proper imaging test is essential. The treatment of choice is complete excision.

## Introduction

Echinococcus species produce hydatidosis, which is a verminosis. Echinococcus vogeli causes the polycystic kind, and the wild dog is the definitive host. Wild canines and rats are the most common hosts [1]. The transit of Echinococcus eggs through the liver and lung is hypothesized to cause hydatid cysts to form in the subcutaneous tissue. Furthermore, subcutaneous tissue involvement can be produced by internal cyst rupture and contamination during surgery, or by disseminated disease, with the creation of a subcutaneous cyst from the rupture of an intra-abdominal cyst that infiltrates the abdominal wall [2-4]. In 1.5 percent of hydatidosis patients, hydatid cysts in subcutaneous tissue have been recorded, ranging from 0.6 percent to 2.6 percent [3]. A 30-year-old woman with a history of hydatid cyst has had her hydatid cyst removed successfully. She had a lump on her abdominal wall when she came in. The diagnosis of hydatid cyst was verified by imaging (computed tomography (CT) and magnetic resonance imaging (MRI)). Before having surgical excision, she was given a course of albendazole. She then finished an albendazole course after surgery.

## Patient and observation

**Patient information:** a 30-year-old woman with a known hydatid cyst. In Yamen, where she lived in 2000, she had a history of liver hydatid illness. She has no recollection of being treated or not. On July 10, 2021, she appeared to the surgery clinic. She had been suffering from abdominal pain and swelling around the umbilicus for the past seven years. She also has a lump in her right lower abdomen, which she says is related to pain that started three months ago. There have been no changes in bowel habits, nausea, or vomiting. There was no fever, no loss of appetite, no weight loss, and no night sweats. There has been no previous hospitalization. A history of CS from 5 years ago was mentioned in previous surgeries. A previous medical history included a diagnosis of hepatic hydatid disease in the year 2000, medical termination of missed miscarriage February 2021.

**Clinical findings:** examination showed that vital signs were within normal range, the patient was alert, conscious, and oriented. The abdomen was soft and lax, no guarding, no rigidity. An infraumbilical hernia was discovered, as well as a 4 x 3 cm bulge on the left flank with neither erythema nor discomfort. Her laboratory results were normal (WBC 5.7, Hb 10.5, coagulation profile normal), and serology was negative.

**Timeline:** on July 10, 2021, the patient came to the surgery clinic. The patient´s medical history and examination were completed. The result of laboratory tests the next day were normal. The diagnosis of a multiloculated cystic mass along the right lower anterior abdominal wall muscles (with cysts inside cysts) and a liver calcified cyst measuring 9 x 8.5 x 4.7 cm was confirmed by ultrasound (US) and CT abdomen. An MRI abdomen was done on July 21, 2021, which yielded the same findings. The patient was taken to the hospital for surgical removal of a hydatid cyst in the abdomen wall on August 15,2021. The patient stayed in the hospital for three days before being discharged in a stable condition on anti-helminthic analgesia on August 19, 2021. She was followed in infectious disease and surgery clinic 1 week after discharge. Both visits were unremarkable.

**Figure 1:**
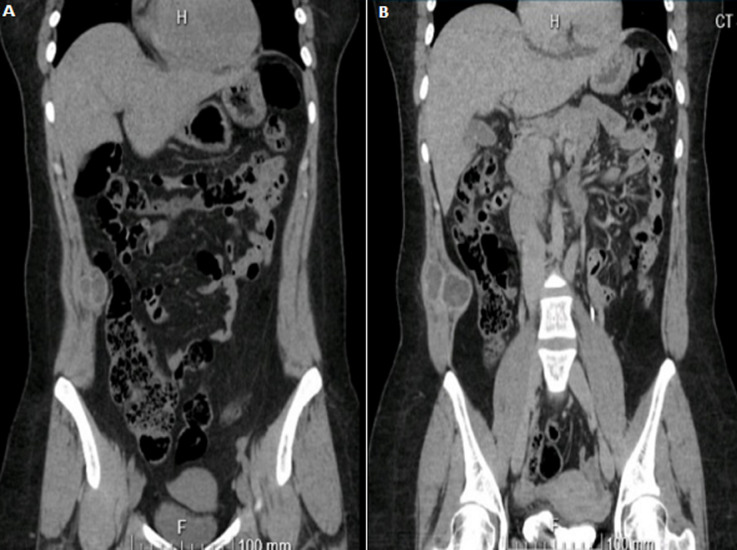
CT abdomen showing venous phase (A), arterial phase (B) a complex abdominal wall mass

**Figure 2:**
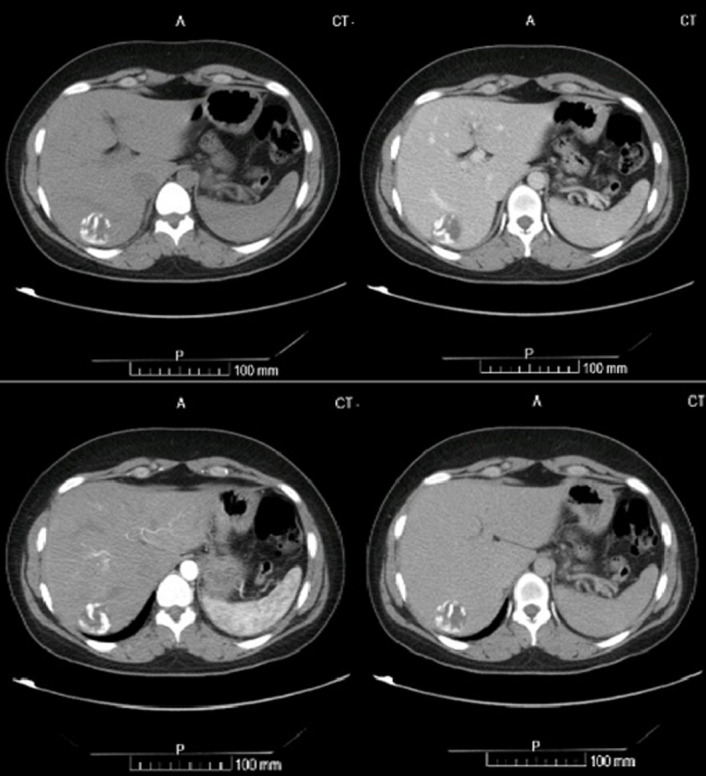
CT abdomen (axial section) showing a calcified hydatid cyst in the liver from left to right: pre-contrast, venous, arterial, delayed phase of the liver mass

**Figure 3:**
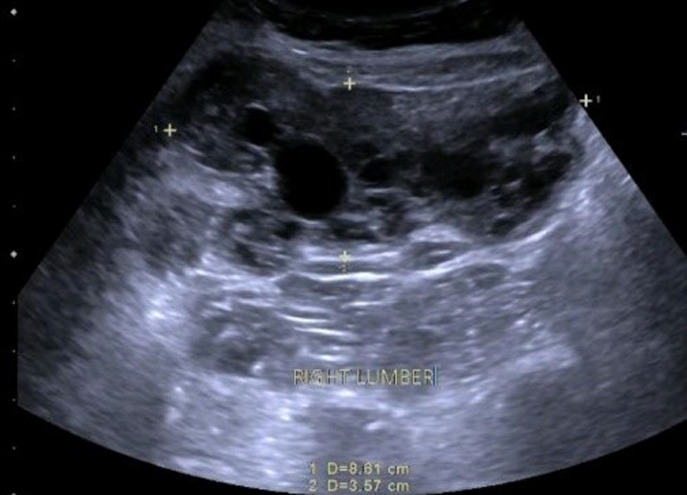
US of right lumbar region showing a heterogenous mixed echogenicity cystic and solid hypoechoic oval macro-lobulated mass lesion between anterior abdominal wall muscles with mostly intramuscular component measuring 8.6 x 3.5 cm

**Diagnostic assessment:** a CT abdomen was performed outside in July 2021 (Figure 1, Figure 2), which revealed a 6 cm hernia, infraumbilical, calcified hydatid cyst in the liver, and a complicated abdominal wall mass? hydatid cyst. An US was performed on 12/July/2021 (Figure 3) and revealed: a heterogeneous mixed echogenicity cystic and solid hypoechoic oval macro-lobulated mass lesion is seen at right lumbar region corresponding to the site of patient concern insinuating itself between anterior abdominal wall muscles with mostly intramuscular component in the right lumbar region corresponding to the site of patient concern insinuating itself between anterior abdominal wall muscles with mostly intramuscular component in Its 8.6 x 3.5 cm in size. A colour Doppler examination revealed no internal vascularity.

**Figure 4:**
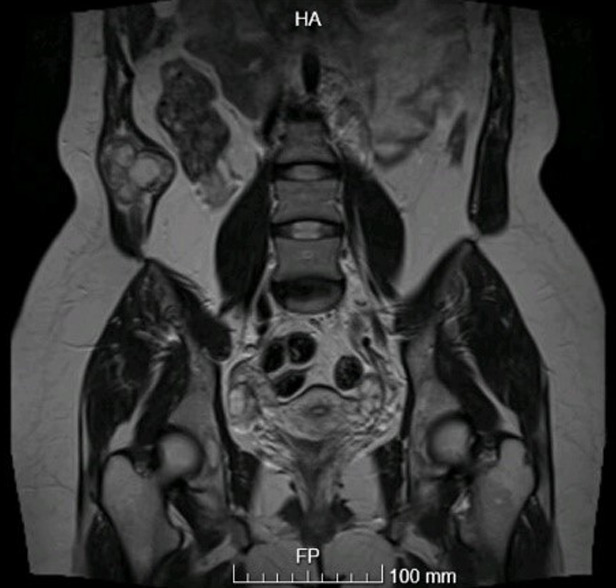
MRI abdomen showing the abdominal wall mass; a multiloculated cystic mass along the right lower anterior abdominal wall muscles (with cysts within cysts) measuring 9 x 8.5 x 4.7 cm

**Figure 5:**
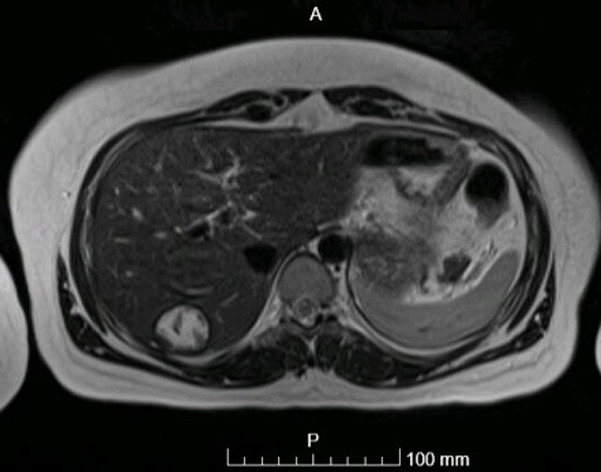
MRI abdomen showing the liver mass with a segment 6/7 cyst with T2 dark rim and dark intra-cystic areas corresponding to calcifications by CT (most probably a calcified hydatic cyst)

The diagnosis was difficult, and the presence of a hydatid cyst had to be confirmed. As a result, on July 20, 2021, an MRI abdomen was performed (Figure 4, Figure 5) and revealed the following: liver: segment 6/7 cyst with T2 dark rim and dark intra-cystic areas corresponding to calcifications by CT. Most likely, this is a calcified hydatic cyst. There are no additional lesions. Otherwise, the liver shows normal signal and shape. A multiloculated cystic tumour is also present along the right lower anterior abdominal wall muscles (with cysts within cysts). This extends into the peritoneum along its most inferior anterior aspect medially after passing through the muscles. However, the cystic tumour is mostly intramuscular. On postcontrast pictures, the mass is rim enhancing with low signal centrally. Along the increasing rim, there is only minor confined diffusion. There are no nodules in the soft tissue. There is no fat in this product. The presence of a hepatic calcified lesion and the formation of cysts inside cysts confirm the diagnosis of hydatid cyst. This mass is 9 x 8.5 x 4.7 cm in size (craniocaudally by antero-posterior by transverse). Umbilical hernia with a small amount of fat is also noted. The final diagnosis was verified to be a hydatid cyst in the abdomen wall.

She was also referred to a clinic for infectious diseases. She was given a two-week regimen of albendazole before to surgery. The patient and her family were advised of the need for surgery, which included mass removal and hernia repair with the addition of two meshes ± component separation. Furthermore, the dangers, benefits, and risks of not undergoing the operation, as well as alternatives, were discussed. The patient agreed to the treatment and gave his approval.

**Therapeutic intervention:** the patient was admitted to the surgery ward on August 15, 2021. Cefazolin 1000 mg IV and metronidazole 500 mg IV STAT were administered to her prior to surgery. Endotracheal intubation was used to perform the procedure under general anaesthetic. The patient was positioned supine. The area was properly prepped and draped. For umbilical hernia, a 4 cm midline incision was made, open knife was used to remove fat and identify the defect, and 2 cm of the content was fat. The hernia sac was removed, tented, and sent to histopathology for analysis. The mesh was secured beneath the abdominal wall. The abdominal wall was closed in a consistent pattern on all layers.

The patient was then placed in a left lateral posture. Over the mass, a transverse incision was made. Blunt and sharp dissection were used for dissection. The cyst was attached to the muscles of the abdominal wall. Cyst and fascia were removed. Part of content came out during the procedure and was suctioned. The sac and abdominal wall were irrigated by hypertonic saline then albendazole suspension. The cyst was removed completely and sent for histopathology. Further irrigation with hypertonic saline was done. Defect was reconstructed using mesh. Then fascia was approximated by PDS. Drain was inserted subcutaneously. Skin was closed by clips. The patient was extubated and transferred to post anaesthesia care unit then regular surgical ward in a good stable condition.

She was started on analgesia (paracetamol 1g TID regular, ibuprofen 400 mg BID regular, tramadol 100 mg PRN TID), metoclopramide 10 mg TID, omeprazole 40 mg once daily. Infectious disease team was consulted regarding hydatid cyst treatment. They started her on albendazole 400 mg PO BID and recommended to continue albendazole 400 mg BID PO for one month postoperatively.

**Follow-up and outcome of interventions:** she was admitted to the hospital and stayed for three days. The hospital stay was uneventful. She reported mild pain in right lower quadrant, but no nausea, no vomiting. No constitutional symptoms. Tolerating orally. She was mobilizing and using the spirometer. Examination showed vitals were within normal range, alert, conscious, oriented. Abdomen was soft and lax She was followed up daily. J vac drain amount decreased daily and was at day 3 postoperatively at amount of 36 ml/day, serous in colour. The patient was sent home with the drain. She was discharged on albendazole 400mg BID PO for 1 month, analgesia (paracetamol 1g/ caffeine 60mg/ codeine phosphate 16mg PRN QID, ibuprofen 400mg PRN BID for 10 days), laxative (senna 45mg/ispagula husk 330 mg/ plantaginis 7800 mg once daily).

One week after surgery, she was seen at an infectious diseases clinic. She was doing well, no complains. Tolerating albendazole 400 mg BID. The patient was advised to complete albendazole for one month (ending on September 20, 2021).

One week after her surgery, she was seen in the surgical clinic. She was in good health and had no complaints. Examination revealed that vital signs were within normal limits, that the patient was aware, conscious, and oriented, that the abdomen was soft and lax, and that the incision was dry and clean. The J vac drain was producing 15-20 mL/day and was therefore removed. One week after surgery, her laboratory workup (CBC, AST, ALT) was within normal range. In addition, the patient was examined at the surgical clinic two weeks after surgery. She was in good health and had no complaints. The wound was found to be clean, and the staplers were removed.

Histopathological examination confirmed the diagnosis of umbilical hernia sac (biopsy 1) and right abdominal wall hydatid cyst (biopsy 2). (Biopsy1) gross description showed single irregular piece of yellowish-brown fatty with rubbery tissue measures 7.1*4.5*1.3 cm. Serial cut sections showed grossly unremarkable tissue surface. (Biopsy 2) gross description showed single irregular piece of greyish dark brown rubbery to firm corrugated tissue measured 10*7.2*2.1 cm. Serial cut sections showed greyish dark brown haemorrhagic tissue surface.

**Patient perspective:** when the patient was examined in the clinic after surgery, she had no complaints, examination was unremarkable. She expressed her sincere gratitude to the surgical team.

**Informed consent:** consent was obtained from the patient for publishing the article.

## Discussion

In the Mediterranean, the Middle and Far East, and South America, hydatid disease is considered as an endemic disease. Direct contact with a dog or eating of foods contaminated by dog excrement are the most common ways for humans to become infected [1]. Single or numerous cysts, uni- or multiloculated cysts, and thin or thick walled cysts are all possible [5]. In 2.3 percent of patients, a hydatid cyst is seen in the subcutaneous tissue without involvement of the liver or lungs. During a physical examination, the most typical finding is a palpable mass. The clinical finding is mainly caused by compression of the affected organ [6]. Hydatid cysts typically grow slowly, with annual growth rates ranging from 1 to 3 cm in diameter [7]. Hydatid illness has a non-specific clinical course. It is determined by the number, size, and location of cysts. Abscess, persistent hematoma, synovial cyst, and cancer should all be checked out as differential diagnoses [8]. Because our patient had previously had a hydatid cyst, hydatid cyst was one of the differentials for abdominal wall mass.

Complete excision of hydatid cyst in the subcutaneous tissue can be an efficient surgical option, where all cysts are radically removed [9,10]. The cyst must be removed completely and avoiding spilling its contents since hazardous anaphylaxis and dissemination have been reported [10]. When radical removal is impossible, surgical treatment is not curative and recurrence is expected. In these cases, supplementary chemotherapy can be started. Antihelmintic drugs, such as mebendazole (50 mg/kg/daily) or albendazole (10 mg/kg/daily) are needed for 4 to 6 months [10]. We have completely surgically excised the cyst; however, it had been ruptured in the operative field. Complete excision of hydatid cysts in the subcutaneous tissue, in which all cysts are radically eliminated, can be an effective surgical alternative [9,10]. Because dangerous anaphylaxis and dispersion have been observed [10], the cyst must be fully excised while avoiding leaking its contents. Surgical treatment is not curative when radical excision is difficult, and recurrence is predicted. Supplementary chemotherapy can be started in these circumstances. For 4 to 6 months, antihelmintic medications such as mebendazole (50 mg/kg/day) or albendazole (10 mg/kg/day) are required [10]. In our case, the cyst had been totally surgically removed, but it had ruptured in the operation field. Thus, the patient was discharged on a complete course of albendazole postoperatively.

The study´s strength is that the hydatid cyst was found in an uncommon place. Imaging confirmed the diagnosis, and the cyst was surgically removed. It ruptured during an operation room excision and was successfully treated with hypertonic saline and albendazole. The study's fault is that a hydatid cyst ruptured in the operating room but was handled adequately. Because subcutaneous hydatid cyst is a rare diagnosis, it's vital to keep it in mind as a possible cause of abdominal wall mass. It also necessitates precise imaging in order to correctly locate and extract it. It should be treated with anti-helminthic medications before surgery to reduce the risk of contamination during the procedure if ruptured.

The fact that a hydatid cyst in subcutaneous tissue is a rare diagnosis is one of our limitations. There had been few cases of its management that had been reported. In addition, there are just a few published studies on the management of ruptured hydatid cysts in the operating room and their outcomes.

## Conclusion

To summarize, a subcutaneous hydatid cyst should be considered as one of the differential diagnoses for soft tissue masses, particularly in patients who have lived in endemic areas. To rule out alternative possibilities, proper imaging is essential. The treatment of choice is complete excision.

## References

[1] D'Alessandro A, Rausch RL (2008). New aspects of neotropical polycystic (Echinococcus vogeli) and unicystic (Echinococcus oligarthrus) echinococcosis. Clinical microbiology reviews.

[2] Prado AS, Castillo P, Gaete F (2006). Hydatid cyst of the scalp. Plastic and reconstructive surgery.

[3] Kayaalp C, Dirican A, Aydin C (2011). Primary subcutaneous hydatid cysts: a review of 22 cases. International journal of surgery (London, England).

[4] Chevalier X, Rhamouni A, Bretagne S, Martigny J, Larget-Piet B (1994). Hydatid cyst of the subcutaneous tissue without other involvement: MR imaging features. AJR, American journal of roentgenology.

[5] Kiresi DA, Karabacakoglu A, Odev K, Karaköse S (2003). Uncommon locations of hydatid cysts. Acta radiologica (Stockholm, Sweden: 1987).

[6] Gossios KJ, Kontoyiannis DS, Dascalogiannaki M, Gourtsoyiannis NC (1197). Uncommon locations of hydatid disease: CT appearances. European radiology.

[7] Sayek I, Tirnaksiz MB, Dogan R (2004). Cystic hydatid disease: current trends in diagnosis and management. Surgery today.

[8] Engin G, Acunas V, Rozanes I, Acunas G (2000). Hydatid disease with unusual localization. European radiology.

[9] Costa D, Coltro PS, Neves CF, Gonçalves H, Ferreira MC, Farina-Junior JA (2021). Surgical treatment of an uncommon hydatid cyst of the abdominal wall. ANZ journal of surgery.

[10] Safioleas M, Nikiteas N, Stamatakos M, Safioleas C, Manti CH, Revenas C, Safioleas P (2008). Echinococcal cyst of the subcutaneous tissue: a rare case report. Parasitology international.

